# Supplementary material for: Proteomic analysis of SARS-CoV-2 particles unveils a key role of G3BP proteins in viral assembly
Source: Nat Commun. 2024 Jan 20;15:640. doi: 10.1038/s41467-024-44958-0 (PMC10799903; doi:10.1038/s41467-024-44958-0)
Supplement: Supplementary file 1 — Supplementary Information [file 41467_2024_44958_MOESM1_ESM.pdf]

SUPPLEMENTARY INFORMATION

Proteomic analysis of SARS-CoV-2 particles unveils a key role of G3BP proteins in viral assembly

Emilie Murigneux[1#], Laurent Softic[1#], Corentin Aubé[1#], Carmen Grandi[2], Delphine Judith[1], Johanna Bruce[3], Morgane LeGall[3], François Guillonneau[3+] , Alain Schmitt[1], Vincent Parissi[4], Clarisse Berlioz-Torrent[1], Laurent Meertens[5], Maike M.K. Hansen[2] and Sarah Gallois-Montbrun[1*]

Supplementary Information associated with this article include:

Supplementary Figs. 1-7

Supplementary Tables 1-5

Supplementary Data 1 (attached dataset)

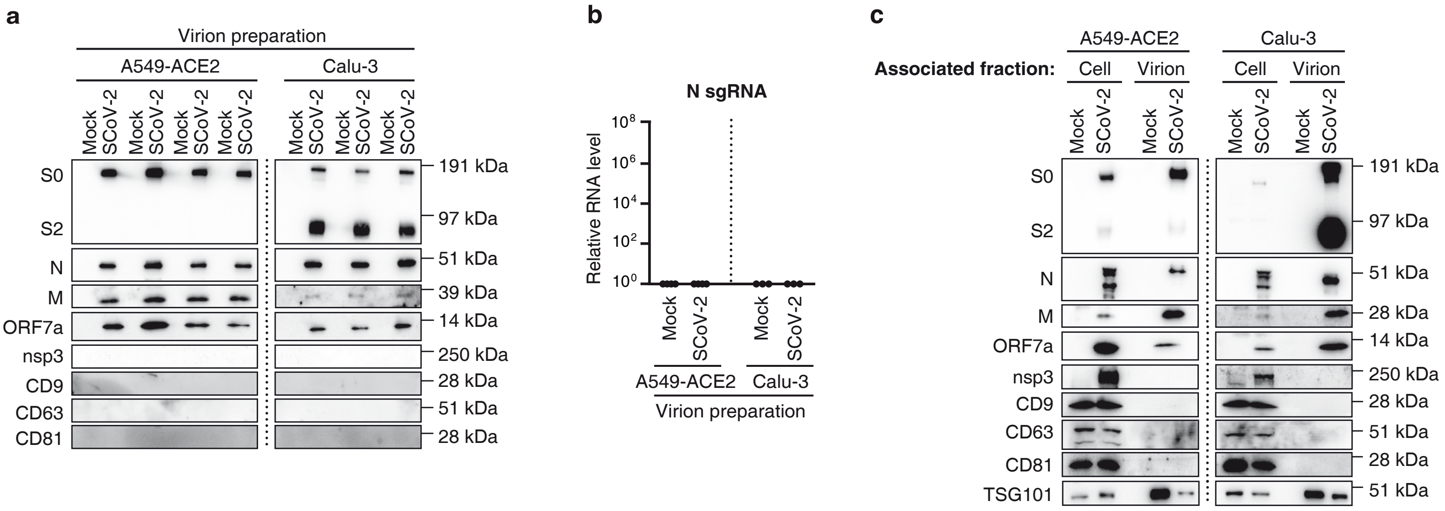

Supplementary Fig. 1 Characterization of virion preparations from A549-ACE2 and Calu-3 cells.
Virion preparations from mock- and SARS-CoV-2-infected A549-ACE2 and Calu-3 cells were isolated as described in Fig. 1. **a** Immunoblotting of all virion preparations related to Fig. 1b. Uncleaved S0 and cleaved S2, N, M, ORF7a and nsp3 viral proteins were detected using indicated antibodies. Exosomal markers CD9, CD63 and CD81 were not detected by immunostaining. **b** SARS-CoV-2 subgenomic N RNA (N sgRNA) was undetected by qPCR in the virion preparations from A549-ACE2 and Calu-3 cells. **c** Detection of viral uncleaved S0, cleaved S2, N, M, ORF7a, nsp3 as well as exosome markers, CD9, CD63, CD81 and TSG101 in cell-associated and in virion-associated fractions of mock- or SARS-CoV-2-infected A549-ACE2 and Calu-3 cells by immunoblotting using indicated antibodies. For analysis, 0.25% of total cell lysates and 2% of ultracentrifuged supernatants were loaded. Source data are provided as a Source Data file.

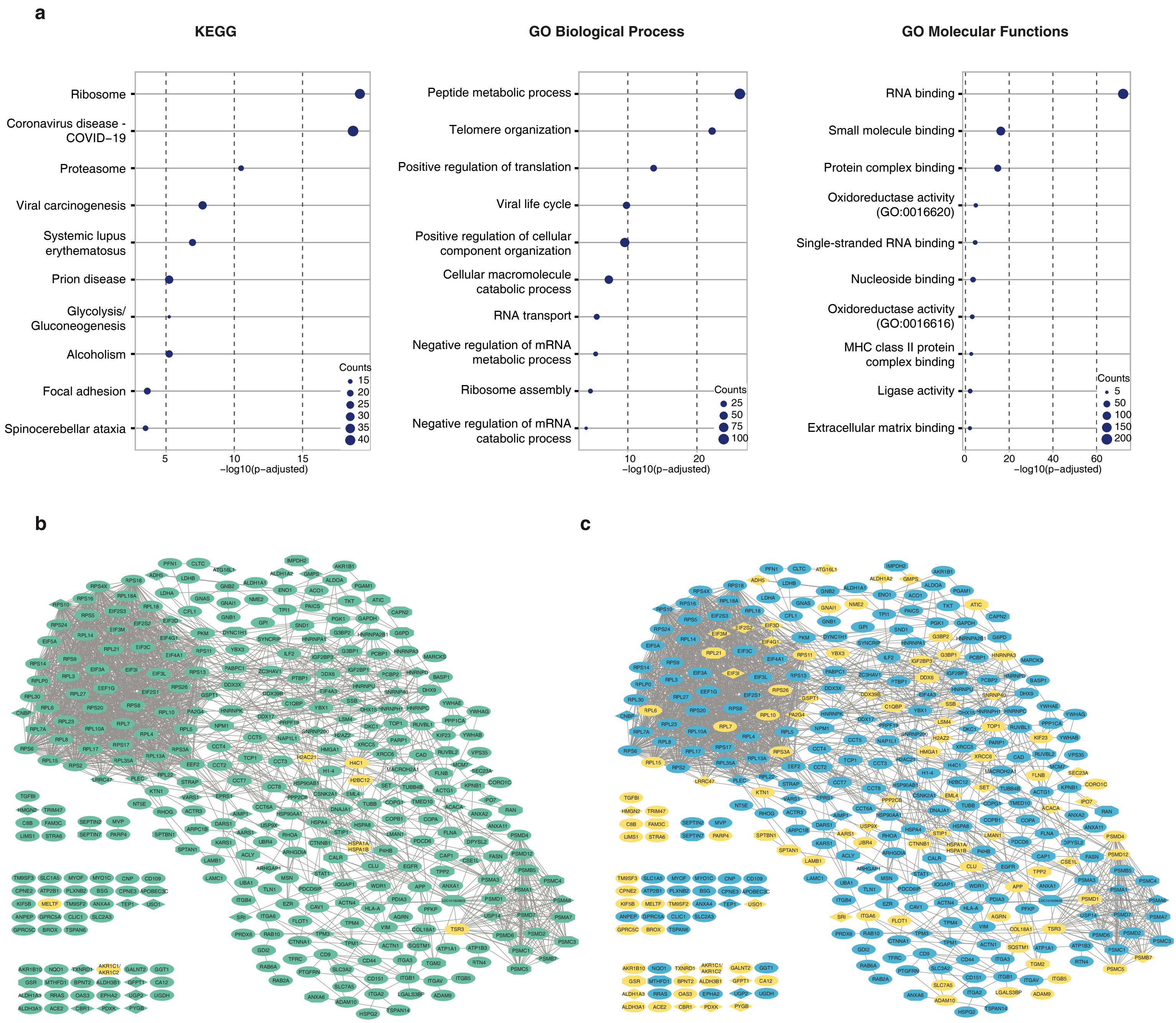

Supplementary Fig. 2 Characterization of cellular factors associated with SARS-CoV-2 virions.
Three hundreds and fifty eight cellular factors associated with SARS-CoV-2 virions produced from A549-ACE2 and Calu-3 cells and isolated by ultracentrifugation on sucrose cushion and ultrafiltration as in Fig 1 were analyzed. **a** KEGG and GO analyses (biological process and molecular functions) of cellular factors associated with SARS-CoV-2 virions. Enrichment terms of the 358 SARS-CoV-2 virion associated factors plotted as a function of -log10 (p-adjusted). The diameter of each blue circle is correlated with the number of proteins (counts) associated with each term. **b** SARS-CoV-2 virion associated factors detected in natural sites of SARS-CoV-2 infection according to Singh et al.[17] are in green. **c** The profile of SARS-CoV-2 virion associated proteins was compared to that of 20 viruses isolated by various technologies, including Ebola Zaire, Marbrug Lake Victoria, Human immunodeficiency Virus type 1, Moloney Murine Leukemia Virus, Epstein Barr, Herpes Simplex Type 1, Pseudorabies Virus, Human Cytomegalovirus, Kaposi's Sarcoma-associated Herpesvirus, Murine gammaherpesvirus 68, Vaccinia Virus, Influenza A, Hepatitis C, Vesicular Stomatitis Virus, Rift Valley Fever Virus, Newcastle Disease Virus, Porcine Reproductive and Respiratory Syndrome Virus, Respiratory Syncytial Virus, Lassa Virus and Sindbis Virus[18, 19]. Factors in common to SARS-CoV-2 and at least one of those viruses are highlighted in blue. Factors highlighted in yellow are exclusively associated with SARS-CoV-2 virions. Source data are provided as a Source Data file.

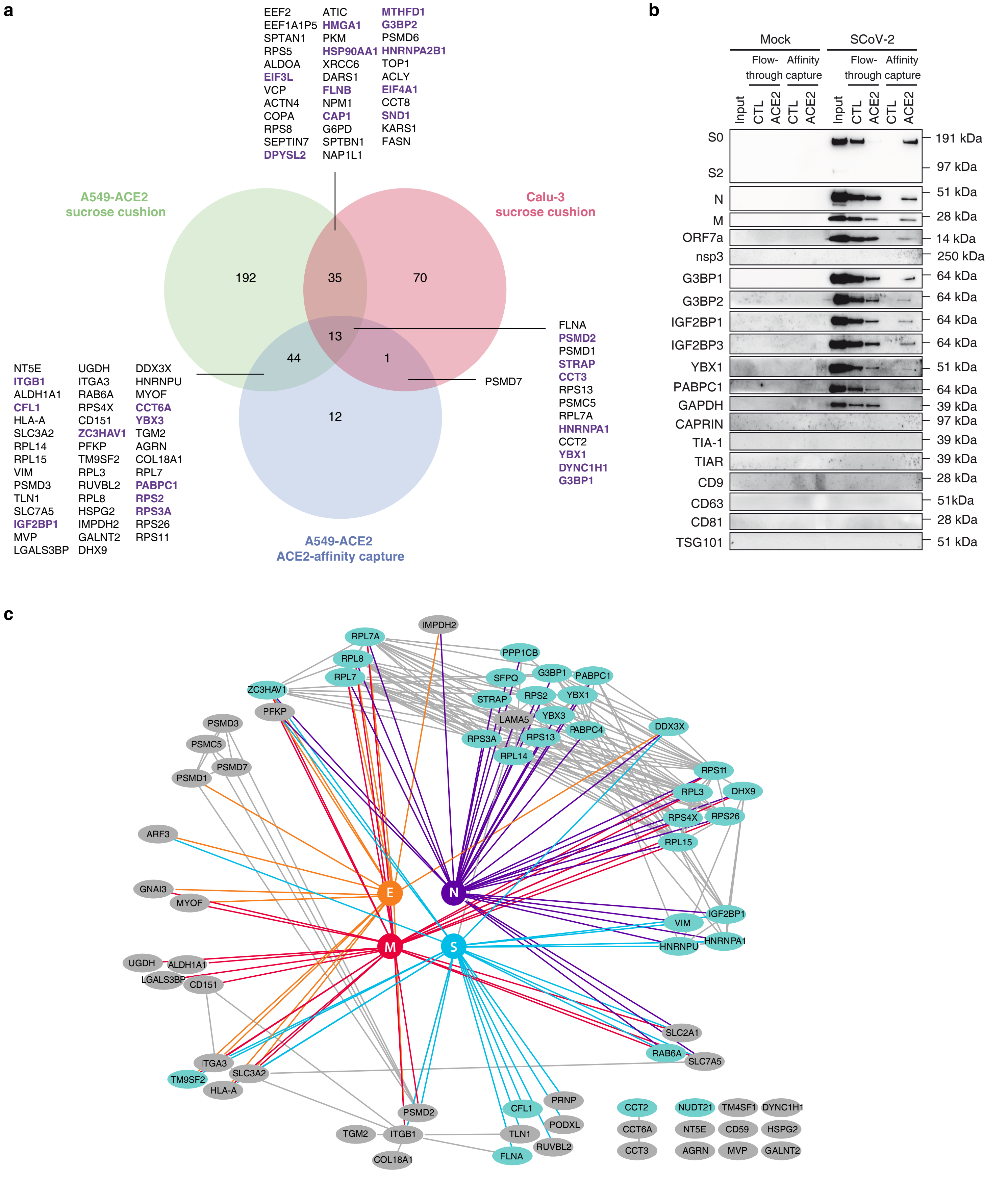

Supplementary Fig. 3 A subset of stress granules factors are associated with SARS-CoV-2 virions.
a Venn diagram highlighting the number of host proteins enriched in SARS-CoV-2 virions isolated by ultracentrifugation on sucrose cushion and ultrafiltration from either A549-ACE2 cells (in green; related to Fig. 1f) or Calu-3 cells (in pink; related to Fig. 1g) or isolated from A549-ACE2 cells using ACE2 affinity capture (in blue; related to Fig. 3d). The proteins common to at least two virion isolation strategies are detailed. Stress granule proteins are highlighted in purple. **b** Proteins in preparations from mock- and SARS-CoV-2-infected A549-ACE2 cells, isolated on ultracentrifugation on sucrose cushion (viral input), in the Flow-through and associated with SARS-CoV-2 virions further isolated by ACE2 affinity capture or in biotin control were detected by immunoblotting with the indicated antibodies. The right part is related to Fig. 3b. The left part is indicative of the absence of proteins in ACE2 affinity capture from mock-infected cells (n=1). **c** Host factors identified with SARS-CoV-2 virions isolated by ACE2 affinity capture were analyzed using String and a protein-protein interaction network was built based on known cellular interactions between host proteins of the network (grey edges) and/or with known interactions with the viral structural proteins (colored edges) as in Fig 2. Purple, red, blue and orange nodes indicate viral N, M, S and E structural proteins, respectively. Nodes highlighted in cyan indicate host proteins known to bind SARS-CoV-2 RNAs in infected cells as in Fig 2. Source data are provided as a Source Data file.

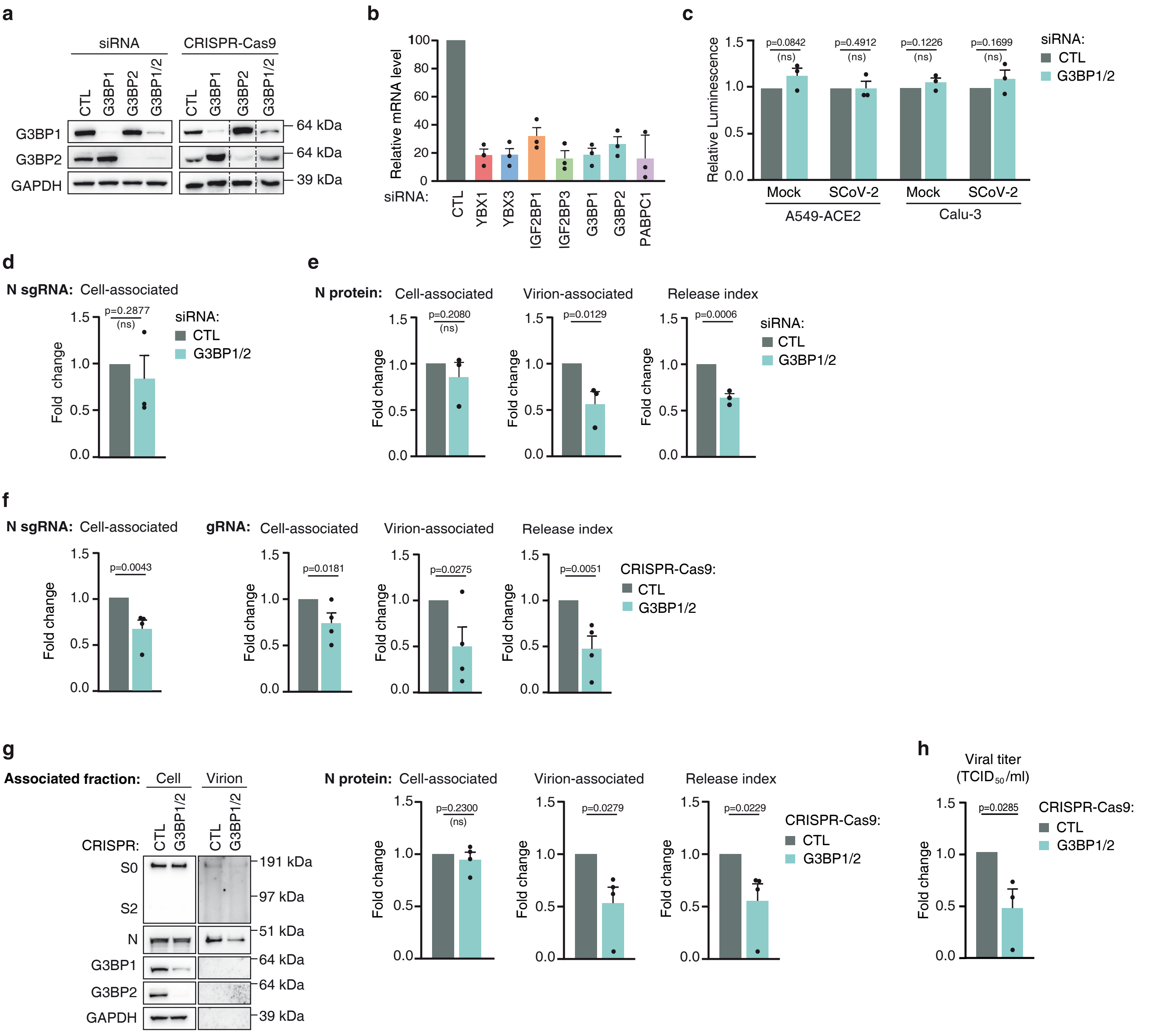

Supplementary Fig. 4 G3BP proteins impact the production of SARS-CoV-2 virions at a late stage of infection.
a Immunoblot analyses of G3BP1 and G3BP2 in A549-ACE2 cells KD after siRNA transfection (left panel) or transiently KO by CRISPR-Cas9 in transduced cell population (right panel) (n=2 independent experiments). **b** mRNA levels of cellular factors in siRNA transfected cells quantified by qPCR using specific primers detailed in Supplementary Table 1 (n=3 independent experiments). **c** Cytotoxicity assessed in A549-ACE2 and Calu-3 cells either CTL or KD for G3BP1 and G3BP2 (G3BP1/2), and either mock- or SARS-CoV-2-infected (MOI 1, 10 h), using the CellTiter-Glo Luminescent Cell Viability Assay (n=3 independent experiments). **d-e** A549-ACE2 cells CTL or KD for G3BP1 and G3BP2 (G3BP1/2) were infected with SARS-CoV-2 (MOI 1, 10 h). **d** Viral N subgenomic RNA (N sgRNA) level was quantified by qPCR in cell-associated fraction and normalized to Actin level (n=3 independent experiments). **e** Cell- and virion-associated viral protein N was quantified by Elisa and the Release index (defined as the ratio of unnormalized virion-associated over unnormalized cell-associated level) of N was calculated (n=3 independent experiments). **f-h** A549-ACE2 cells transduced with CRISPR-Cas9 CTL or targeting both G3BP1 and G3BP2 (G3BP1/2) were infected with SARS-CoV-2 (MOI 1, 10 h). In all subsequent analyses, identical volumes were used between CTL and G3BP1/2 KO conditions for cell and virion-associated fractions. **f** Cell-associated N sgRNA level normalized to Actin level as well as cell-associated (normalized to Actin level), unnormalized virion-associated level and Release index (as defined as in Fig. 4) of gRNA are shown (n=4 independent experiments). **g** Cell- and virion-associated viral proteins N and S were detected by immunoblotting with the indicated antibodies. A representative image of 4 independent experiment is shown. Nucleocapsid (N) quantification of the cell-associated level normalized to GAPDH level, the unnormalized virion-associated level and the Release index (as defined in Fig. 4) are shown (n=4 independent experiments). **h** Viral titers of the virion-associated fractions were quantified by Tissue Culture Infectious Dose (TCID50) assays (n=3 independent experiments). Data are presented as mean values +/- SEM; One-tailed unpaired Student's t-test was applied. Source data are provided as a Source Data file.

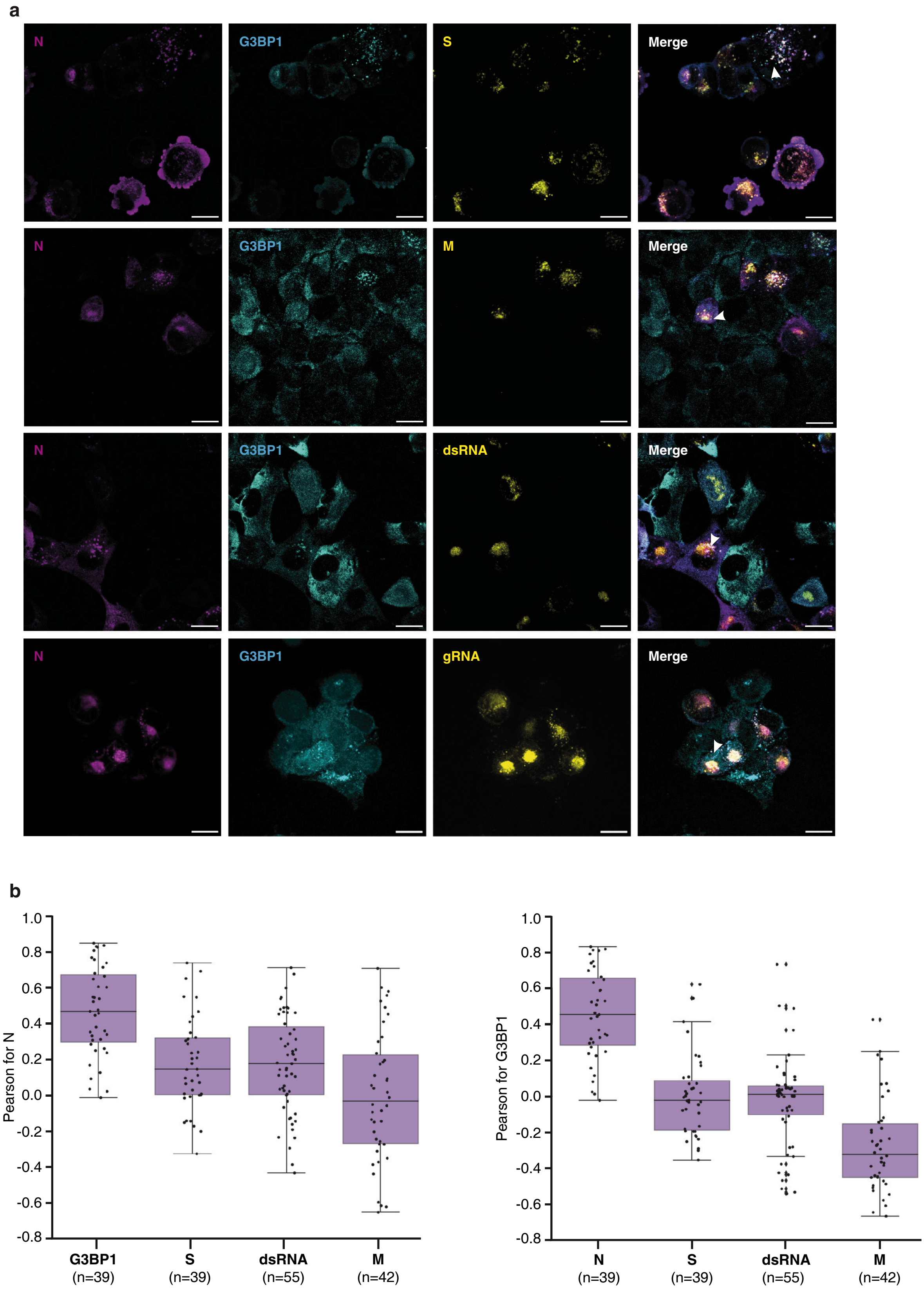

Supplementary Fig. 5 Cellular localization of G3BP1 with virions components in SARS-CoV-2 infected cells at 24 h post-infection.
a Representative confocal images of A549-ACE2 cells infected with SARS-CoV-2 (MOI 0.01) at 24 hpi. Several stages of infection were identified based on the absence or presence of N granules. Cells with N granules analyzed in Fig. 5a-d are indicated with a white arrow, scale bar, 15 µm (n=3 independent experiments for S, M and dsRNA; and n=2 for gRNA). **b** Colocalization between G3BP1, N, S and M in infected cells (24 hpi) was determined using the Pearson's coefficient method. Pearson's coefficients were measured in over 35 cells per condition, with exact numbers indicated in the figure for each condition. Source data are provided as a Source Data file.

a

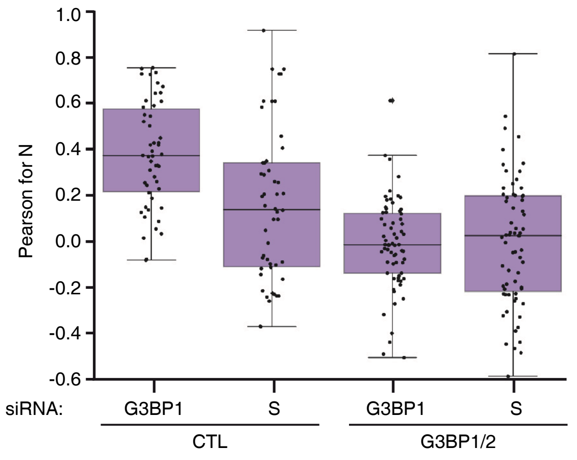

Supplementary Fig. 6 Pearson coefficient of N with G3BP1 and S in control or downregulated cells for both G3BP1 and G3BP2. Colocalization between N and G3BP1 or S in infected cells (10 hpi), related to Fig. 6a, was determined using the Pearson's coefficient method. Pearson's coefficients were measured in n=48 cells for the CTL condition and n=65 cells for the G3BP1/2 KD condition (n=2 independent experiments). Source data are provided as a Source Data file.

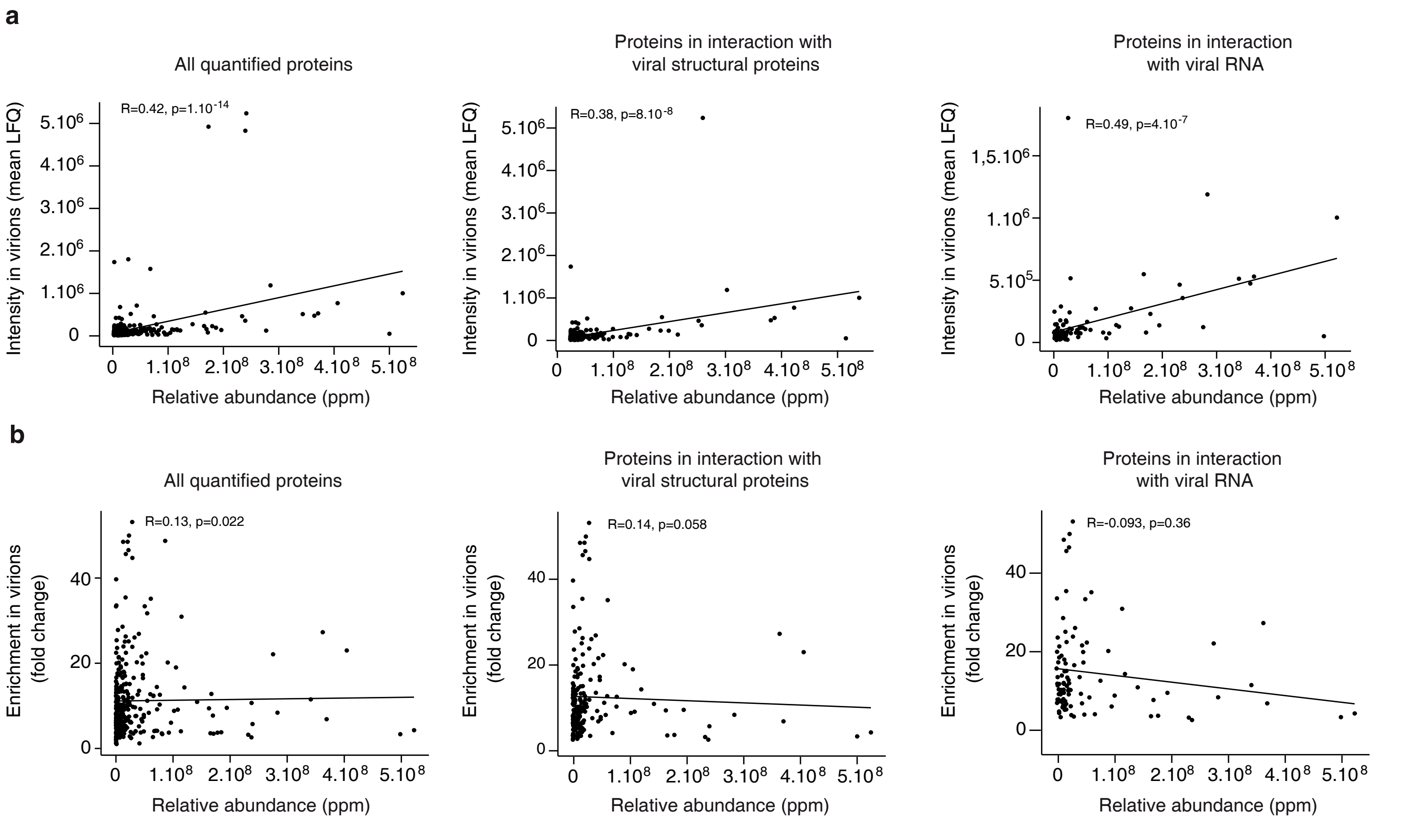

Supplementary Fig. 7 Correlation of signal intensities and enrichment of virion-associated factors with their abundance in whole cell lysate. **a** LC-MS signal intensities (LFQ) of virion-associated factors from four independent experiments were plotted against signal intensities in the total cell lysate from one of the replicates. Details of all virion-associated factors (left), virion-associated factors interacting with viral structural proteins (middle), and virion-associated factors interacting with viral RNA (right) are provided. **b** Enrichement of factors associated with virions compared to control preparation from mock-infected cells from n=4 independent replicates were plotted against signal intensities in the total cell lysate from one of the replicates. Details of all virion associated factors (left), virions-associated factors interacting with viral structural proteins (middle) and virion-associated factors interacting with viral RNA (right) are detailed. Spearman correlations were calculated using the GG Scatter Package from the GG PubR library (version 0.6.0.999) in R. Source data are provided as a Source Data file.

Oligonucleotides	Sequence
SARS-CoV-2 genomic	Fwd : 5’-ACAGGTACGTTAATAGTTAATAGCGT-3’<br>Rev : 5’-ATATTGCAGCAGTACGCACAC A-3’
SARS-CoV-2 subgenomic N	Fwd : 5’-GTTCTCTAAACGAACAAACTAA-3’<br>Rev : 5’-GGCTTGACTGCCGCCTCTGCTCC-3’
Actin	Fwd : 5’-CCCTGGACTTCGAGCAAGAG-3’<br>Rev : 5’-ACTCCATGCCGAGGAAGGAA-3’
YBX1	Fwd : 5’-GAGAAGTGATGGAGGGTGCTGA-3’<br>Rev : 5’-TCTAGGCTGTCTTTGGCGAG-3’
YBX3	Fwd : 5’-ATCCCAACAGAATACAGGCTGG-3’<br>Rev : 5’-GGCGGTAAGTTGGATTTCGATG-3’
IGF2BP1	Fwd : 5’-TAGCTCCTTTATGCAGGCTCC-3’<br>Rev : 5’-GAGAGCTGTTTGATGTGCTGC-3’
IGF2BP3	Fwd : 5’-TGCACGGGAAACCCATAGAAG-3’<br>Rev : 5’-AAACTATCCAGCACCTCCCAC-3’
G3BP1	Fwd : 5’-TACTATCCTCGGTGCTGTGGT-3’<br>Rev : 5’-TTGGTCAATTCAACCTGGGGG-3’
G3BP2	Fwd : 5’-AGCAATAAGAACCCGCCGCA-3’<br>Rev : 5’-GCTGCACAATGTCAAATGCTCC-3’
PABPC1	Fwd : 5’-CCAACCCTGTAATCAACCCCTAC-3’<br>Rev : 5’-CTAGGAGGATAGTATGCAGCACG-3’

Supplementary Table 1: Sequences of qPCR primers used in this study.

Antibodies	Source	Identifier	Application
GAPDH Mouse Monoclonal antibody (0411)	Santa Cruz	sc-47724	WB (1:1000)
SARS-CoV-2 Nucleoprotein Mouse Monoclonal antibody (05)	Sino biological	40143-MM05	WB (1:5000)
SARS-CoV-2 (COVID-19) Nucleocapsid Rabbit Polyclonal antibody	Genetex	GTX135357	IF (1:1000)<br>IP (1µg/assay)
SARS-CoV/SARS-CoV-2 (COVID-19) Spike Mouse Monoclonal antibody (1A9)	Genetex	GTX632604	WB (1:1000)<br>IF (1:1000)
SARS Membrane Protein Rabbit Polyclonal Antibody	Novus Biologicals	NB-100-56569	WB (1:500)
SARS-CoV-2 Membrane Mouse Monoclonal Antibody (1041508)	R&D Systems	MAB10690	IF (1:60)
SARS-CoV-2 (COVID-19) nsp3 Rabbit Polyclonal antibody	Genetex	GTX135589	WB (1:2000)
SARS-CoV/SARS-CoV-2 (COVID-19) ORF7a Mouse Monoclonal antibody (3C9)	Genetex	GTX632602	WB (1:1000)
YBX1 Rabbit Polyclonal antibody	Proteintech	20339-1-AP	WB (1:1000)
IGF2BP1 Rabbit Polyclonal antibody	Proteintech	22803-1-AP	WB (1:1000)
IGF2BP3 Rabbit Polyclonal antibody	Proteintech	14642-1-AP	WB (1:5000)
G3BP1 Rabbit Polyclonal antibody	Proteintech	13057-2-AP	WB (1:5000)
G3BP2 Rabbit Polyclonal antibody	Proteintech	16276-1-AP	WB (1:2000)
PABPC1, PABP Rabbit Polyclonal antibody	Proteintech	10970-1-AP	WB (1:1000)
TIA-1 Rabbit Polyclonal antibody	Proteintech	12133-2-AP	WB (1:1000)
TIAR (D32D3) XP® Rabbit Polyclonal antibody	Cell Signaling Technology	CST8509	WB (1:1000)
CAPRIN1 Rabbit Polyclonal antibody	Proteintech	15112-1-AP	WB (1:1000)
Anti-CD9 Monoclonal Mouse Antibody (MM2/57)	Sigma-Aldrich	CBL162	WB (1:1000)
CD81 Rabbit Polyclonal antibody	Genetex	GTX101766	WB (1:500)
CD63 Goat Polyclonal Antibody	Santa Cruz	sc-7080	WB (1:500)
Purified Anti-TSG101 Mouse Monoclonal antibody (51/TSG101)	BD Biosciences	BD612697	WB (1:1000)
TFR-2 Mouse Monoclonal antibody (B-6)	Santa Cruz	sc-65882	WB (1:1000)
Coralite 594 G3BP1 Mouse Monoclonal antibody (1E4A2)	Proteintech	CL594-66486	IF (1:400)
Rabbit Polyclonal IgG	Diagenode	C1541026	IP (1µg/assay)
SARS-CoV-2 Nucleocapsid Alexa Fluor® 405 Rabbit Monoclonal Antibody (4D0J7)	Novus Biological	NBP3-05764AF405	IF (1:100)
SARS-CoV-2 Spike AlexaFluor® 594 Mouse Monoclonal antibody (CR3022)	Novus Biological	NBP3-12017AF594	IF (1:100)
Anti-dsRNA Mouse Monoclonal antibody (rJ2)	Sigma-Aldrich	MABE1134	IF (1:60)
G3BP1 XP® Rabbit Monoclonal AlexaFluor® 488 conjugate (E9G1M)	Cell Signaling Technology	94496S	IF (1:100)
ERGI3/ERGIC-3 Rabbit Polyclonal Antibody,Cyanine 5 Conjugated	Bioss	bs-13103R-Cy5	IF (1:300)
Polyclonal Rabbit Anti-Mouse Immunoglobulins/HRP	Dako	P0260	WB (1:5000)
Polyclonal Swine Anti-Rabbit Immunoglobulins/HRP	Dako	P0217	WB (1:5000)
Goat anti-mouse IgG (H+L) Alexa Fluor® 594	Abcam	Ab1510116	IF (1:1000)
Donkey anti-mouse IgG (H+L) Alexa Fluor® 488	Invitrogen	A21202	IF (1:1000)
Donkey anti-rabbit IgG (H+L) Alexa Fluor® 647	Invitrogen	A31573	IF (1:1000)

Supplementary Table 2: List of antibodies used in this study.

Oligonucleotides	Sequence	Source	Identifier
Pool of siRNA targeting YBX1	CUGAGUAAAUGCCGGCUUA<br>CGACGCAGACGCCCAGAAA<br>GUAAGGAACGGAUAUGGUU<br>GCGGAGGCAGCAAAUGUUA	Dharmacon	L-010213-00
Pool of siRNA targeting YBX3	GGAAGGGAGUCGUUACGCU<br>GAUGAAGGAUGGAGUCCCA<br>GCAAUAAAGUGGAAGACUA<br>GCACGGAAGACAAGAGAGU	Dharmacon	L-015793-00
Pool of siRNA targeting IGF2BP1	CGAAACACCUGACUCCAAA<br>UGAAGGCCAUCGAAACUUU<br>GAAAGUAGAAUUACAAGGA<br>GCUUAGAGAUUGAACAUUC	Dharmacon	L-003977-00
Pool of siRNA targeting IGF2BP3	CCAAGUGGUUGUCAAAAUA<br>UCGAGGCGCUUUCAGGUAA<br>GGAUUUCAGUUAGAGAAUU<br>GAUAUUGCUUCUAUGAAUC	Dharmacon	L-003976-00
Pool of siRNA targeting G3BP1	GUGGUGGAGUUGCGCAUUA<br>AGACAUAGCUCAGACAGUA<br>GAAGGCGACCGACGAGAUA<br>GCGAGAACAACGAAUAAAU	Dharmacon	L-012099-00
Pool of siRNA targeting G3BP2	UGAAUAAAGCUCCGGAAUA<br>GAAUUUAAGUCUGGGACGA<br>ACAACGACCUAGAGAACGA<br>GCGAUGGUCUUGACUAUUA	Dharmacon	L-015329-01
Pool of siRNA targeting PABPC1	CAUGUAAGGUGGUUUGUGA<br>GAGCAAGGAAACGUAAUUU<br>GGACAAAUCCAUUGAUAAU<br>UGGAUGAGAUGAACGGAAA	Dharmacon	L-019598-00
Pool of ON-TARGETplus Non-targeting Control	UGGUUUACAUGUCGACUAA<br>UGGUUUACAUGUUGUGUGA<br>UGGUUUACAUGUUUUCUGA<br>UGGUUUACAUGUUUUCCUA	Dharmacon	D-001810-10-50

Supplementary Table 3: References and sequences of ON-TARGETplus siRNA used in this study.

Oligonucleotides	Sequence	Source
CRISPR-Cas9 sgRNA G3BP1	AAGCCAGCAGATGCAGTCTA	Eurofins
CRISPR-Cas9 sgRNA G3BP2	CATGGTGGAGTAGATGCTAG	Eurofins

Supplementary Table 4: Sequences of CRISPR-Cas9 guides used in this study.

Probes sequences	Source
tgagttggacgtgtgttttc	BioSearch Technologies
gcgaacctgtaaaacaggca	BioSearch Technologies
ccataacatgaccatgaggt	BioSearch Technologies
tactgaatgccttcgagttc	BioSearch Technologies
aatgcactcaagagggtagc	BioSearch Technologies
ccagttgttcggacaaagtg	BioSearch Technologies
tttcagaacgttccgtgtac	BioSearch Technologies
tggtgacgcaactggataga	BioSearch Technologies
gaaaggcacatttggttgca	BioSearch Technologies
tggctttaacaaaatcgccc	BioSearch Technologies
agtaaccacaagtagtggca	BioSearch Technologies
tccaaaggcaatagtgcgac	BioSearch Technologies
cctgtatggttacaacctat	BioSearch Technologies
cattaagaccttcggaacct	BioSearch Technologies
actgaacaacaccacctgta	BioSearch Technologies
tcaaggacgggtttgagttt	BioSearch Technologies
accttccttaaacttctctt	BioSearch Technologies
gaatgtctgaacactctcct	BioSearch Technologies
acttctgtgggaagtgtttc	BioSearch Technologies
acagcttcactagtaggttg	BioSearch Technologies
acaaactggtgtaccaacca	BioSearch Technologies
atcatattaggtgcaagggc	BioSearch Technologies
aagtaacctttgttggtgca	BioSearch Technologies
gtcttgttgaccaacagttt	BioSearch Technologies
cttatttaaggctcctgcaa	BioSearch Technologies
cacagcagttaaaacaccct	BioSearch Technologies
ttggcacttttctcaaagct	BioSearch Technologies
tacatagccaagtggcattg	BioSearch Technologies
ctagttgtgtagattgtcca	BioSearch Technologies
acacttttatcacctctctt	BioSearch Technologies
tctaggtggaatgtggtagg	BioSearch Technologies
atgtcattgacatgtccaca	BioSearch Technologies
gctccatccaaataagttgg	BioSearch Technologies
gtggtagtactcaaaagcct	BioSearch Technologies
gggtatttccactttttagt	BioSearch Technologies
cttgtagagcaggtggatta	BioSearch Technologies
ccacaagttttacacaccac	BioSearch Technologies
accacactggtaattaccag	BioSearch Technologies
gtaaagcaccgtctatgcaa	BioSearch Technologies
cttgcgtttggatatggttg	BioSearch Technologies
gtatacaccaggtatttggt	BioSearch Technologies
ctggttttagatcttcgcag	BioSearch Technologies
aagcagcggttgagtagatt	BioSearch Technologies
acaaccgtctacaacatgca	BioSearch Technologies
tacattcgactcttgttgct	BioSearch Technologies
ctttacctccattagcatag	BioSearch Technologies
gtactaccagcacagaatgt	BioSearch Technologies
agtgacaagtctctcgcaac	BioSearch Technologies

Supplementary Table 5: smFish probes sequences used in this study.
