# Supplementary material for: Proteomic analysis of SARS-CoV-2 particles unveils a key role of G3BP proteins in viral assembly
Source: Nat Commun. 2024 Jan 20;15:640. doi: 10.1038/s41467-024-44958-0 (PMC10799903; doi:10.1038/s41467-024-44958-0)
Supplement: Supplementary file 3 — Description of Additional Supplementary Files [file 41467_2024_44958_MOESM3_ESM.pdf]

## Description of Additional Supplementary File:

**Supplementary Data 1: Analysis of mass spectrometry data performed on SARS-CoV-2 virions produced from A549-ACE2 and Calu-3 cells, isolated either via ultracentrifugation on a sucrose cushion or through ACE-2 affinity capture.**

For each identified protein, the protein name and ID, the statistical analysis, fold change enrichment as compared to control and the LFQ with or without imputation are presented, as well as comparison of our data with other published datasets (Fig. 2b and Supplementary Figs. 2b, 2c and 3a).
