# Supplementary material for: Proteomic analysis of SARS-CoV-2 particles unveils a key role of G3BP proteins in viral assembly
Source: Nat Commun. 2024 Jan 20;15:640. doi: 10.1038/s41467-024-44958-0 (PMC10799903; doi:10.1038/s41467-024-44958-0)
Supplement: Supplementary file 5 — Reporting Summary [file 41467_2024_44958_MOESM5_ESM.pdf]

nature portfolio

Corresponding author(s): Sarah Gallois-Montbrun

Last updated by author(s): Dec 18, 2023

Reporting Summary

Nature Portfolio wishes to improve the reproducibility of the work that we publish. This form provides structure for consistency and transparency in reporting. For further information on Nature Portfolio policies, see our Editorial Policies and the Editorial Policy Checklist.

Statistics

For all statistical analyses, confirm that the following items are present in the figure legend, table legend, main text, or Methods section.

n/a	Confirmed	
☐	☒	The exact sample size (*n*) for each experimental group/condition, given as a discrete number and unit of measurement
☐	☒	A statement on whether measurements were taken from distinct samples or whether the same sample was measured repeatedly
☐	☒	The statistical test(s) used AND whether they are one- or two-sided<br>*Only common tests should be described solely by name; describe more complex techniques in the Methods section.*
☒	☐	A description of all covariates tested
☒	☐	A description of any assumptions or corrections, such as tests of normality and adjustment for multiple comparisons
☐	☒	A full description of the statistical parameters including central tendency (e.g. means) or other basic estimates (e.g. regression coefficient) AND variation (e.g. standard deviation) or associated estimates of uncertainty (e.g. confidence intervals)
☐	☒	For null hypothesis testing, the test statistic (e.g. *F*, *t*, *r*) with confidence intervals, effect sizes, degrees of freedom and *P* value noted<br>*Give P values as exact values whenever suitable.*
☒	☐	For Bayesian analysis, information on the choice of priors and Markov chain Monte Carlo settings
☒	☐	For hierarchical and complex designs, identification of the appropriate level for tests and full reporting of outcomes
☐	☒	Estimates of effect sizes (e.g. Cohen's *d*, Pearson's *r*), indicating how they were calculated

Our web collection on statistics for biologists contains articles on many of the points above.

Software and code

Policy information about availability of computer code

Data collection	For mass spectrometry: Maxquant version 1.6.17;<br>For imaging on IXplore spinning disk: Olympus cellSens Dimension
Data analysis	Mass spectrometry LFQ: Maxquant version 1.6.17 (https://www.maxquant.org/), PTXQC (Bielow et al, 2026) and Perseus software v1.6.15 (https://maxquant.net/perseus/).<br>Protein-protein interaction networks were built using STRING v.11.5 (https://string-db.org/) and visualized using Cytoscape V.3.8.2. (https://cytoscape.org/);<br>Gene ontologies over-representation analyses were performed with DAVID online tool, updated version 2021 (https://david.ncifcrf.gov/) and plotted with RStudio (version 2023.06.1) using ggplot2 package (version 3.3.5).<br>Images were analyzed using ImageJ2 version 2.9.0/1.53t (https://imagej.net/).<br>Statistics and graphing were performed using GraphPad Prism 8 software (https://www.graphpad.com/scientific-software/prism/).<br>Mander's coefficients of single cells were calculated using in-house Python programs available upon request.<br>smFISH probes were designed using the online tool from Biosearch Technologies (https://www.biosearchtech.com)

For manuscripts utilizing custom algorithms or software that are central to the research but not yet described in published literature, software must be made available to editors and reviewers. We strongly encourage code deposition in a community repository (e.g. GitHub). See the Nature Portfolio guidelines for submitting code & software for further information.

Data

Policy information about availability of data

All manuscripts must include a data availability statement. This statement should provide the following information, where applicable:

- Accession codes, unique identifiers, or web links for publicly available datasets
- A description of any restrictions on data availability
- For clinical datasets or third party data, please ensure that the statement adheres to our policy

All relevant data supporting the key findings of this study and any associated accession codes and references are available within the article and in the Supplementary Information Files or from the corresponding authors upon request. Source data are provided with this paper.
The mass spectrometry proteomics data generated in this study have been deposited in the ProteomeXchange Consortium via the PRIDE partner repository under accession code PXD038321, PXD045406, PXD045409.

Research involving human participants, their data, or biological material

Policy information about studies with human participants or human data. See also policy information about sex, gender (identity/presentation), and sexual orientation and race, ethnicity and racism.

Reporting on sex and gender	N/A
Reporting on race, ethnicity, or other socially relevant groupings	N/A
Population characteristics	N/A
Recruitment	N/A
Ethics oversight	N/A

Note that full information on the approval of the study protocol must also be provided in the manuscript.

Field-specific reporting

Please select the one below that is the best fit for your research. If you are not sure, read the appropriate sections before making your selection.

☒ Life sciences ☐ Behavioural & social sciences ☐ Ecological, evolutionary & environmental sciences

For a reference copy of the document with all sections, see nature.com/documents/nr-reporting-summary-flat.pdf

Life sciences study design

All studies must disclose on these points even when the disclosure is negative.

Sample size	No sample size calculation was performed. Our experiments involved infection in cultured cells where sample size is not relevant. We performed experiments with at least 50,000 cells per infection experiment. Details to that are given in the publication (Bartenschlager, 2023).
Data exclusions	We excluded experiments where the controls did not work. No predetermined exclusion criteria was established.
Replication	Experimental findings were reliably reproduced through at least 3 to 4 repeated independent experiments (as indicated in the figure legends) except for Fig. 6c where N and N/S puncta were quantified in 2 independent experiments (10 images per conditions per experiment and a total number of at least 500 cells). All attempts at replication were successful
Randomization	Randomization is not applicable in this study as no animal or clinical samples were involved.
Blinding	In most of the study, investigators were not blinded as the readouts were not subjective.<br>For imaging data, no blinding was required because the possibility of biases affecting interpretation of results was managed by means of controls and statistical analysis.

Reporting for specific materials, systems and methods

We require information from authors about some types of materials, experimental systems and methods used in many studies. Here, indicate whether each material, system or method listed is relevant to your study. If you are not sure if a list item applies to your research, read the appropriate section before selecting a response.

Materials & experimental systems

n/a	Involved in the study
☐	☒ Antibodies
☐	☒ Eukaryotic cell lines
☒	☐ Palaeontology and archaeology
☒	☐ Animals and other organisms
☒	☐ Clinical data
☒	☐ Dual use research of concern
☒	☐ Plants

Methods

n/a	Involved in the study
☒	☐ ChIP-seq
☒	☐ Flow cytometry
☒	☐ MRI-based neuroimaging

Antibodies

Antibodies used	anti-GAPDH mouse monoclonal (clone 0411, Santa Cruz, catalog #SC-47724, lot number #H2521);<br>anti-SARS-CoV-2 Nucleoprotein / NP mouse monoclonal (clone 05, Sino biological, catalog #40143-MM05, lot number #HB14JL0605);<br>anti-SARS-CoV-2 nucleocapsid rabbit polyclonal (Genetex, catalog #GTX135357, lot number #43979),<br>anti-SARS-CoV/SARS-CoV-2 spike mouse monoclonal (clone 1A9, Genetex, catalog #GTX632604, lot number #44174),<br>anti-SARS Membrane Protein rabbit polyclonal (Novus Biologicals, catalog #NB-100-56569, lot number #IN021405D-7);<br>anti-SARS-CoV-2 Membrane mouse monoclonal (clone 1041508, R&D Systems, catalog # MAB10690, lot number #CNPU012081);<br>anti-SARS-CoV-2 nsp3 Rabbit Polyclonal antibody (Genetex, catalog #GTX135589, lot number #44062);<br>anti-SARS-CoV/SARS-CoV-2ORF7a Mouse Monoclonal antibody (clone 3C9, Genetex catalog #GTX632602, lot number #42219)<br>anti-YB1 rabbit polyclonal (Abcam, catalog #Ab12148, lot number #00046294);<br>anti-IGF2BP1 rabbit polyclonal (Proteintech, catalog #22803-1-AP, lot number #00045767);<br>anti-IGF2BP3 rabbit polyclonal (Proteintech, catalog #14642-1-AP, lot number #00048003);<br>anti-G3BP1 rabbit polyclonal (Proteintech, catalog #13057-2-AP, lot number #00103096);<br>anti-G3BP2 rabbit polyclonal (Proteintech, catalog #16276-1-AP, lot number #00079746);<br>anti-PABPC1 rabbit polyclonal (Proteintech, catalog #10970-1-AP, lot number #00085769);<br>anti-TIA-1 rabbit polyclonal antibody (Proteintech, catalog # 12133-2-AP, lot number #00094740);<br>anti-TIAR (D32D3) XP® Rabbit Polyclonal antibody (Cell Signaling Technology, catalog #CST8509, lot number #2);<br>anti-CAPRIN1 Rabbit Polyclonal antibody (Proteintech, catalog #15112-1-AP, lot number #00073970);<br>anti-CD9 Monoclonal Mouse Antibody (clone MM2/57, Sigma-Aldrich, catalog #15112-1-AP, lot number #2897163);<br>anti-CD81 Rabbit Polyclonal antibody (Genetex, catalog #GTX101766, lot number #42270);<br>anti-CD63 Goat Polyclonal Antibody (Santa Cruz, catalog # sc-7080, lot number #A089);<br>anti-TSG101 Mouse Monoclonal antibody (clone 51/TSG101, BD Biosciences, catalog #BD612697, lot number #52841);<br>anti-TFR-2 Mouse Monoclonal antibody (clone B-6), Santa Cruz, catalog #sc-65882, lot number #L1113);<br>anti-G3BP1 Coralite 594 mouse monoclonal (clone 1E4A2, Proteintech, catalog #CL594-66486, lot number #21002130);<br>anti-SARS-CoV-2 Nucleocapsid (ARC5077-01-02) Alexa Fluor® 405 (clone 4D0J7, Novus Biological, catalog #NBP3-05764AF405, lot number #D105494);<br>anti-SARS-CoV-2 Spike Alexa Fluor® 594 mouse monoclonal (clone CR3022, Novus Biological, catalog #NBP3-12017AF594, lot number #D101358);<br>anti-dsRNA mouse monoclonal (clone rJ2, Sigma-Aldrich, catalog #MABE1134, lot number #3768054);<br>anti-G3BP1 XP Alexa Fluor® 488 conjugate rabbit monoclonal (clone E9G1M, Cell Signaling Technology, catalog #94496S, lot number #1);<br>anti-ERRGI3/ERGIC-3 Cy5 conjugated rabbit polyclonal (Bioss, catalog# bs-13103R-Cy5, lot number #BB0414540);<br>Rabbit Polyclonal IgG (Diagenode, catalog #C1541026, lot number #RIG001AM);<br>Polyclonal Rabbit Anti-Mouse Immunoglobulins/HRP (Dako, catalog #P0260, lot number #41543857);<br>Polyclonal Swine Anti-Rabbit Immunoglobulins/HRP (Dako, catalog #P0217, lot number #41386888);<br>Goat anti-mouse IgG (H+L) Alexa Fluor® 594 (Abcam, catalog #Ab1510116, lot number #1918277);<br>Donkey anti-mouse IgG (H+L) Alexa Fluor® 488 (Invitrogen, catalog #A21202, lot number #2428531);<br>Donkey anti-rabbit IgG (H+L) Alexa Fluor® 647 (Invitrogen, catalog #A31573, lot number #1903516).
Validation	All primary antibodies used for Western Blot have been validated by the manufacturers according to the information on their websites: Santa Cruz https://www.scbt.com; Sino biological: https://www.sinobiological.com/antibodies/; GeneTex: https://www.genetex.com/; Novus Biologicals: https://www.novusbio.com/; Proteintech: https://www.ptglab.com/<br>Cell Signaling Technology: https://www.cellsignal.com/products/antibody-conjugates/; Sigma-Aldrich: https://www.sigmaaldrich.com/; BD Biosciences: https://www.bdbiosciences.com/en-eu.<br><br>All primary antibodies used for immunoprecipitation have been validated by the manufacturers according to the information on their websites:<br>GeneTex: https://www.genetex.com/; Diagenode: https://www.diagenode.com/en/categories/all-antibodies.<br><br>Unconjugated primary antibodies and conjugated antibodies used for immunofluorescence have been validated by the manufacturers according to the information on their websites:

GeneTex: https://www.genetex.com/; R&D Systems: https://www.rndsystems.com/; Proteintech: https://www.ptglab.com/; Novus Biologicals: https://www.novusbio.com/; Sigma-Aldrich: https://www.sigmaaldrich.com/; Cell Signaling Technology: https://www.cellsignal.com/products/antibody-conjugates/; Bioss https://www.biossusa.com/products/
Anti-SARS-CoV-2 Nucleocapsid Alexa Fluor® 405 (Novus Biological, catalog #NBP3-05764AF405) used for immunofluorescence was validated in the laboratory in SARS-CoV-2-infected cells versus mock-infected cells.

Eukaryotic cell lines

Policy information about cell lines and Sex and Gender in Research

Cell line source(s)	Vero-E6 cells (African green monkey kidney cells, ATCC CRL-1586), Calu-3 (Human lung adenocarcinoma, ATCC HTB-55), and HEK293T (human embryonic kidney cells, ATCC CRL-3216) cell lines are from ATCC.<br>A549-ACE2 cells (human tumorigenic lung epithelial cells) described in Labeau et al. (Cell Reports, 2022) were provided by Ali Amara's laboratory.<br>The parental A549 and double knock-out (ΔG3BP, clone 5) cells were kindly provided by Dr Sun Hur (Paget et al., Molecular Cell, 2023).
Authentication	Commercial cell lines have been authenticated by ATCC.<br>Cells provided by other laboratories were authenticated by their morphology.
Mycoplasma contamination	We are regularly testing mycoplasma contaminations, and our cells are free from mycoplasma.
Commonly misidentified lines (See ICLAC register)	No commonly misidentified cell lines were used in this study.

Plants

Seed stocks	N/A
Novel plant genotypes	N/A
Authentication	N/A
